# ADHD-AID: Aiding Tool for Detecting Children’s Attention Deficit Hyperactivity Disorder via EEG-Based Multi-Resolution Analysis and Feature Selection

**DOI:** 10.3390/biomimetics9030188

**Published:** 2024-03-20

**Authors:** Omneya Attallah

**Affiliations:** 1 Department of Electronics and Communications Engineering, College of Engineering and Technology, Arab Academy for Science, Technology and Maritime Transport, Alexandria 21937, Egypt; o.attallah@aast.edu; 2 Wearables, Biosensing and Biosignal Processing Laboratory, Arab Academy for Science, Technology and Maritime Transport, Alexandria 21937, Egypt

**Keywords:** electroencephalogram (EEG), attention deficit hyperactivity disorder (ADHD), discrete wavelet transform, variational mode decomposition, empirical wavelet decomposition, machine learning

## Abstract

The severe effects of attention deficit hyperactivity disorder (ADHD) among adolescents can be prevented by timely identification and prompt therapeutic intervention. Traditional diagnostic techniques are complicated and time-consuming because they are subjective-based assessments. Machine learning (ML) techniques can automate this process and prevent the limitations of manual evaluation. However, most of the ML-based models extract few features from a single domain. Furthermore, most ML-based studies have not examined the most effective electrode placement on the skull, which affects the identification process, while others have not employed feature selection approaches to reduce the feature space dimension and consequently the complexity of the training models. This study presents an ML-based tool for automatically identifying ADHD entitled “ADHD-AID”. The present study uses several multi-resolution analysis techniques including variational mode decomposition, discrete wavelet transform, and empirical wavelet decomposition. ADHD-AID extracts thirty features from the time and time–frequency domains to identify ADHD, including nonlinear features, band-power features, entropy-based features, and statistical features. The present study also looks at the best EEG electrode placement for detecting ADHD. Additionally, it looks into the location combinations that have the most significant impact on identification accuracy. Additionally, it uses a variety of feature selection methods to choose those features that have the greatest influence on the diagnosis of ADHD, reducing the classification’s complexity and training time. The results show that ADHD-AID has provided scores for accuracy, sensitivity, specificity, F1-score, and Mathew correlation coefficients of 0.991, 0.989, 0.992, 0.989, and 0.982, respectively, in identifying ADHD with 10-fold cross-validation. Also, the area under the curve has reached 0.9958. ADHD-AID’s results are significantly higher than those of all earlier studies for the detection of ADHD in adolescents. These notable and trustworthy findings support the use of such an automated tool as a means of assistance for doctors in the prompt identification of ADHD in youngsters.

## 1. Introduction

Attention deficit hyperactivity disorder (ADHD), as it is currently defined, understood, and managed, is a syndrome that is independent of etiology or anything other than the presence of symptoms of attention deficits and/or hyperactivity [1]. About 60 percent of children experience signs into their adult years. The associated nervous systems and irregular cognitive functioning play a major role in the development of ADHD [2]. Furthermore, some environmental factors are responsible for the development of ADHD including the consumption of drugs, addiction to alcohol, and smoking during pregnancy [3]. Adolescents with ADHD have trouble concentrating on a given item or duty for an extended period, have behavioral changes, and exhibit hyperactive impulse control issues, which negatively affect how well they are able to learn and interact with others [4]. Later or untreated ADHD raises associated risks such as poor social skills, retarded education, subpar academic performance, low self-esteem, a greater likelihood of attempting acts of violence, and injury vulnerability [5]. Kids with ADHD can complete daily tasks while discovering new things with the assistance of early identification, reliable evaluation, and appropriate medications.

Initial identification of ADHD is crucial for the individual’s recovery. Clinical psychiatrists as well as psychologists are typically the ones who diagnose ADHD in adolescents. Clinical assessment is accomplished through several approaches involving scales/questionnaires [6], interviews with parents along with the children themselves [7], as well as certain ongoing performance examinations [8]. To diagnose ADHD, it is essential to collect symptom history from parents and teachers in various environments, ensuring they meet the DSM criteria for duration and frequency. Additionally, it is crucial to eliminate other conditions that may resemble ADHD such as depression, sleep deprivation, etc. Despite the existence of various methods, the assessment takes a prolonged period [9], requires an elevated degree of clinical proficiency, and can occasionally be inaccurate. The bias inherent in such strategies also calls into doubt the accuracy of the detection. Furthermore, according to a review [10], numerous medical professionals said they did not have the sufficient familiarity necessary to identify ADHD. Thus, another perspective from a computational standpoint such as artificial intelligence to automate the process could additionally increase the medical professional’s trustworthiness regarding the result of their assessment and could additionally conserve a significant amount of time. Although artificial intelligence has been extensively used in the medical domain to aid the detection and diagnosis of various diseases [11,12,13,14], it may face challenges in real-world EEG recordings due to noise and uncertainties. Comprehensive training on enormous databases is essential for algorithms to effectively manage complexities, as noted by the reviewer. Regrettably, extensive datasets of ADHD-related EEG recordings in natural settings may not be easily accessible at this time.

Several studies have been conducted regarding developing aiding tools based on artificial intelligence technology to speed up and improve the precision of the entire procedure of detecting neurological and neurodevelopment diseases [15,16,17,18]. The latest research has focused on initial ADHD recognition via several brain imaging modalities such as electroencephalography (EEG) [19], magnetoencephalography [20], magnetic resonance imaging [21], functional magnetic resonance imaging [22], and others. Among all these approaches, the EEG method has emerged as one of the most popular methods for diagnosing ADHD [23]. This is due to its accessibility, informational value, and affordability. EEG waves are commonly used to identify physiological and brain anomalies including epileptic attacks, emotions, fatigue, and stress [24]. EEG is also used with assistive technology for patients with motor disabilities. EEG data can monitor alterations in brain function caused by ADHD [25,26]. However, complex-level structures in the complex records generated by the brains of humans are challenging to identify [27]. With the help of machine learning (ML), detecting these complicated patterns is achievable. Many studies have employed machine learning approaches to analyze EEG signals and detect ADHD [6,23,28,29,30,31].

### 1.1. Research Gaps

Numerous studies did not examine the most appropriate electrode placement on the skull, which impacts the identification process. Other studies extracted features from a single domain, either time, frequency, or time–frequency. Some studies extracted a few features. Numerous research articles employed one or a few feature extraction approaches to obtain features. Many studies did not perform a feature selection (FS) procedure to select the most significant features, thus reducing the feature space dimension, which lowers the complexity of the training models. Others relied on deep learning models with high complexity and a huge number of parameters. Some studies tested their model on private datasets. Few of them depended on a few participants. Multiple research articles achieved relatively low accuracy; therefore, their models could not be deemed reliable for identifying ADHD.

### 1.2. Contributions and Novelty

This study introduces an automated tool for detecting ADHD based on several machine-learning approaches. To overcome the previous limitations raised in the literature, this study adopts numerous multi-resolution analysis approaches to analyze EEG waves and eliminate noise. Since feature extraction is the key element for the successful classification of EEG waves, the present study has obtained thirty features (nonlinear features, band-power features, entropy-based features, and statistical features) from time and time–frequency domains to detect ADHD. Furthermore, this study examines the finest EEG electrode placement for ADHD detection. It also investigates the best combination of locations that impact detection accuracy. Additionally, it exhibits several FS techniques to select the most influential features that affect ADHD diagnosis, thus lowering the complexity and training duration of the classification.

The key contributions and originality of the present study are summarized as follows:

- Extracting features from multiple domains including, time and time–frequency, and then combining them instead of relying on a single domain.
- Utilizing several multi-resolution analysis methods to analyze EEG signals and remove noise such as discrete wavelet transform (DWT), variational mode decomposition (VMD), and empirical wavelet transform (EWT).
- Employing multiple feature extraction approaches such as nonlinear features, band-power features, entropy-based features, and statistical features.
- Exploring the best electrode placement site that influences the identification performance.
- Introducing various FS approaches to select the highly significant features, thus diminishing the complexity of the classification models.

### 1.3. This Paper’s Structure

The remaining sections of this paper are structured as follows. The second section presents the related works on ADHD detection using EEG signals and ML/DL techniques. The third section describes the methods and the proposed tool entitled ADHD-AID. Subsequently, the forth section explains the parameters established. Next, the fifth section presents the results, followed by section six which discusses the main findings of the results and the limitations of ADHD-AID, and compares the results of ADHD-AID with other studies in the literature. Lastly, the final section concludes the paper.

## 2. Related Works

Numerous EEG-based research articles have been carried out throughout recent years to recognize the presence of ADHD [18,19,20]. The complicated details generated by human brains make it challenging to find significant features and structures. There are two categories of ADHD detection frameworks, which are the traditional ML-based frameworks and DL-based frameworks. This section discusses both frameworks.

### 2.1. ML-Based Frameworks

ML approaches have been working to identify patterns for decades, with the goal of predicting and categorizing EEG data. However, feature extraction is the main element for the successful identification and classification of brain waves. Several studies have employed multiple feature extraction and machine learning approaches; among them, the research article [21] employed three empirical decomposition approaches including EWT, empirical mode decomposition (EMD), and empirical variational decomposition (EVD). The authors extracted 15 features from each domain of the EWT, EMD, and EVD independently. Next, they applied a genetic algorithm and neighbor component analysis to each set of extracted features from EWT, EMD, and EVD individually. The selected features were then fed into a support vector machine classifier (SVM) and artificial neural networks (ANNs) to identify ADHD in children. Alternatively, the study [22] utilized variational mode decomposition (VMD) followed by Hilbert transform to decompose EEG signals and then obtained 41 statistical and nonlinear features from the fifth decomposed mode of the signal. Afterward, features entered the explainable boosted machine model to identify ADHD. Moreover, the study [4] proposed a new approach referred to as variational mode and Hilbert transform-based EEG rhythm separation and employed several ML classifiers reaching an accuracy of 99.95%.

In addition, the study [6] attained power spectral density features, entropy-based features, and bi-spectral features. The authors then adopted minimum redundancy and maximal relevance (mRMR) FS to select features. Those selected features were then used to train an SVM classifier. Conversely, the researchers of [23] directly used the EEG data obtained from 19 channels as inputs to three machine learning classifiers. These researchers investigated the impact of different brain regions on the performance of the training models. On the contrary, the study [24] extracted 10 statistical, power spectral density, and entropy-based features from the time domain. Then, the principal component analysis approach was used to reduce features that enter an SVM classifier to detect ADHD and differentiate it from healthy cases. Meanwhile, the study [25] utilized intrinsic time-scale decomposition (ITD). A number of connectivity-based features were extracted using different mixtures of extracted features from a single domain of the modes, which ITD created. Afterward, these features were used as inputs to several classifiers such as SVM, k-nearest neighbor (k-NN), naïve Bayes, decision trees, and bagging ensembles. In contrast, in the study [26], brain waves were used to create effective connectivity matrices. By measuring the directed phase transfer entropy among every couple of the channels, the effective connectivity matrices of all of them were determined to form a feature matrix. These features were then reduced using a genetic algorithm, and then the chosen features were used to learn an ANN. Elsewhere, the study [27] explored the relationship between ADHD and visuospatial problems, which involve challenges in processing visual information. This study examined 16-year-old adolescents with ADHD and analyzed their brain activity using EEG during a visual processing task, comparing it to a control group. The subsequent findings were stated: Children with ADHD exhibited unique brain activity patterns when analyzing intricate visual stimuli in comparison to the control group. The disparities were most evident in the lower frequency brainwave bands, specifically delta and theta. The authors suggested that these particular EEG characteristics could serve as potential indicators for detecting visual processing challenges linked to ADHD. This study indicated that EEG could be a useful instrument for comprehending the fundamental mechanisms of visual processing issues in children with ADHD. The results could help enhance the creation of better diagnostic methods and treatments for ADHD.

On the other hand, some studies have employed different FS approaches for selecting features extracted from EEG signals to detect ADHD. Among them, the studies [19,28] used the least absolute shrinkage and selection operator FS approach, the study [29] used the Relief FS approach, the paper [30] employed PCA, hybrid step-wise regression, ridge regression, and correlation-based FS, and the paper [24] employed the ANOVA FS method.

### 2.2. DL-Based Frameworks

Other studies have employed DL models for identifying ADHD; among them, in the article [31], the authors used two recurrent networks involving long short-term memory networks and gated recurrent networks and averaged their results. The research article [32] converted EEG signals into RGB images using frequency bands and then fed a CNN consisting of 13 layers to identify ADHD. On the other hand, the article [33] employed connectivity-based features as inputs to a customized CNN. Similarly, the study [34] employed dynamic connectivity analysis to obtain features that then fed a convolutional long short-term memory network model that used an attention mechanism. On the contrary, the article [35] employed EEGNet to identify ADHD without any pre-processing or feature extraction steps. Meanwhile, the study [36] employed VMD and robust local mode decomposition to obtain features that were then utilized to train a CNN model. The study [37] extracted frequency features using Fourier transform (FT), and then these features were fed into a customized CNN which employed a Layer-wise Relevance Propagation procedure for channel selection. Table 1 summarizes the literature on ADHD identification using EEG signals.

### 2.3. Limitations of Previous Frameworks

Table 1 highlights the main limitations of each study. Numerous research articles in the literature have demonstrated some difficulty in obtaining undiscovered details of EEG waves [31]. Limited articles employed feature extraction from EEG data directly without applying a pre-filtering or denoising approach, while some feature extractors such as entropy, Lyapunov exponent, and fractal dimension methodologies generated poor results because of noise and improper scaling range choice. The appropriate values for filter coefficients are crucial for the EEG analysis-based methods of filtering to produce sharp filtering limits. Fast Fourier transform (FFT) has problems with noise sensitivity, time–frequency localization, subpar spectrum calculation, and incorrectly localized peaks [32]. Despite CNN-based methods offering the ability to extract and categorize features at the same time, they necessitate greater amounts of memory. Additionally, the currently used methods select their ML models based on empirical data. When numerous testing configurations are used, a single ML algorithm cannot assure the same performance. Furthermore, the majority of previous studies employed one method for EEG analysis. Moreover, they obtained features from either time, frequency, or time–frequency domains. Nevertheless, employing features from numerous domains and several analysis methods could improve performance [33,34]. To overcome the previously mentioned limitations, this study proposes a framework for ADHD detection based on EEG signals, multiple multi-resolution analyses, and FS approaches. It extracts features from multiple domains including time and time–frequency, and then combines them instead of relying on a single domain. In addition, it utilizes several multi-resolution analysis methods to analyze EEG signals and remove noise such as DWT, VMD, and EWT. Furthermore, ADHD-AID employs multiple feature extraction approaches such as nonlinear features, band-power features, entropy-based features, and statistical features.

## 3. Materials and Methods

### 3.1. Multi-Resolution Analysis

The nonlinear behavior, complexity, and nonstationary nature of the EEG wave make it challenging to recognize and analyze in its original state. The decomposition of an EEG wave into its multiple components is necessary to obtain comprehensive knowledge and indicative features of the signal. Each method of analysis has its unique advantages; thus in this study, multiple multi-resolution analysis approaches are employed and compared.

#### 3.1.1. Variational Mode Decomposition

The original input is divided by VMD into a pre-determined amount of components with distinct sparsity characteristics. Every mode’s frequency domain bandwidth is selected as its level of sparsity. The procedures listed below can be employed for accessing each mode’s bandwidth: (1) computing each mode’s unilateral range of frequencies using the Hilbert transform, (2) shifting each mode’s frequency range into “baseband” by its determined center frequency, and (3) utilizing squared L2 norm to calculate the bandwidth, resulting in the subsequent restriction issue [41].

(1) minhm,wm∑m∂t∂t+jπt∗hmte−jwmt22 ∑mhm=z  

[22]

where *M* is the overall amount of modes, *w_m_* is the frequency that corresponds to the *m*th mode, and *z*(*t*) is the original signal. Utilizing Lagrangian multipliers ($\vec{\lambda }$) and the quadratic penalty factor ($\hat{a}$), the restricted problem is transformed into an unrestricted one. This results in the addition of $\vec{\lambda }$ and $\hat{a}$ for improved convergence properties. The enhanced Lagrangian is represented by the following equation [41]:
  (2) Lhm,wm,λ→=a^∑m∂t∂t+jπt∗hmte−jwmt22+zt−∑mhmt22+λ→t,zt−∑mhmt                                                                              

[22]

#### 3.1.2. Discrete Wavelet Transform

One well-liked method for transforming a signal that is discrete in time into its wavelet representations, which demonstrates time–frequency details, is the discrete time wavelet transform (DWT) [42]. In the DWT, there are in fact a number of wavelets that can be generally categorized as orthogonal wavelets. The invention of the perpendicular form is credited to Hungarian mathematician Alfréd Haar [43]. The signal (*X*) enters a low-pass filter with an impulse response (*L*) in the DWT investigation, which results in a convolution process, illustrated below.

(3) $$DWT\left[n\right]=\left(X*L\right)\left[n\right]=\sum_{k=-\infty }^{\infty }X\left[n\right]L\left[n-k\right]$$

[44]

where *k* is the instance/sample and *n* is the decomposition level.

**Figure 1:**
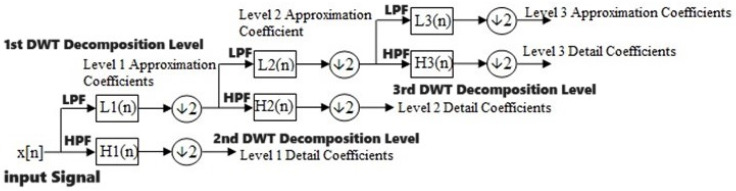
DWT multi-resolution analysis [45].

The input data are also subjected to the high-pass filter (*H*). The result is divided into two sections: the detailed coefficients (*D*_1_), which come from the high-pass filter, and the approximation coefficients (*A*_1_), which come from the low-pass filter [44]. The Nyquist rule states that 50% of an input’s spectrum is removed. Therefore, the output of the low-pass filter result in Figure 1 is decreased by two, and it is then analyzed again by crossing it through additional high and low-pass filters, in which *L* and *H* are the low and high-pass filters’ impulse responses, respectively, while every single one of them is reduced by two, as is apparent here:

(4) $${DWT}_{low}\left[n\right]=\sum_{k=-\infty }^{\infty }X\left[n\right]L\left[2n-k\right]$$

[44]

(5) $${DWT}_{high}\left[n\right]=\sum_{k=-\infty }^{\infty }X\left[n\right]H\left[2n-k\right]$$

[44]

#### 3.1.3. Empirical Wavelet Decomposition

The empirical wavelet transform (EMT) is a traditional completely adaptive signal analysis method employed to analyze nonstationary data by choosing the right range of frequency for band-pass filters. The extremely complicated EEG signal is subjected to the EWT in order to be divided into detail coefficients (*D*_1_) that stand for high-frequency components and approximation coefficients (*A*_1_) that provide low-frequency components. In order to identify the frequency band with the most useful information, the approximation coefficient (*A*_1_) is once again divided into detail coefficient (*D*_2_) and approximation coefficient (*A*_2_). The EWT is used for the decomposition of the EEG signals into an array of band-limited modes and comprises the following steps:

Step 1: To obtain the frequency spectrum associated with the EEG waveform with the desired frequency interval [0, π], FFT is applied to the input EEG data signals.

Step 2: To determine the edge frequency ranges {Ω_j_}_j=0,1,2,…*n*_, the EEG signals are divided into n portions using the horizon identification approach. The frequency of the edge Ω_0_ = 0 and Ω_n_ = π. As a result, the frequency spectrum is written as [0, Ω_1_], [Ω_1_, Ω_2_],….[Ω_n−1_, π].

Step 3: To acquire capable coefficients from the segmented EEG portions, the EWT function and the EWT scaling function are utilized.

To count *n* segments, the captured time-domain EEG signal *X*(*t*) is considered. Utilizing local maxima of *X*(*t*), the proper number of segments is determined, and the Fourier spectrum of frequency [0, π] for each of them is assigned as ${ʌ}_{n}=\left[{Ω}_{n},{Ω}_{n}\right]$. One must take into consideration the period of transition that revolves around each center, which is taken as the one in which the inner product of the applied signal and the wavelet function are used for calculating the detail coefficients.

### 3.2. EEG Dataset

**Figure 2:**
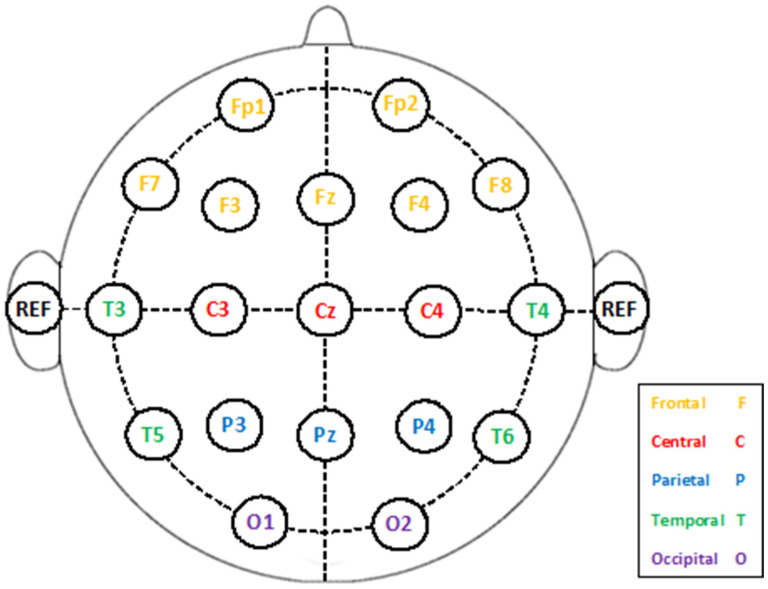
Electrode placement on the participants’ skull [48].

The dataset “EEG data for ADHD/Control children” on IEEE Dataport [38] provides an important resource for academics studying EEG activity in children with ADHD in comparison to healthy controls. The dataset consists of recordings from 121 participants, 61 of whom have been diagnosed with ADHD (48 boys and 13 girls), with an average age of 9.62 ± 1.75 years, and 60 are healthy controls (50 boys and 10 girls) with an average age of 9.85 ± 1.77 years [46]. The participants are all between the ages of 7 and 12 years. Qualified psychiatrists verified the ADHD diagnoses according to DSM-IV criteria. The other participants were normal controls who did not have any neuropsychological disorders. The standard 10–20 system was used to collect EEG data from 19 electrode positions (Fp1, Fp2, Fz, Cz, Pz, C3, T3, T4, F3, F4, F7, F8, P3, P4, T5, T6, O1, O2, Fp1, Fp2, Fz, Cz, and Pz). Figure 2 shows the position placement of these electrodes. The EEG recordings were performed with a standardized 19-channel system at a sampling frequency of 128 Hz to ensure uniformity and enable in-depth analysis of brain wave patterns. Subjects engaged in a visual attention task during the recordings, which required them to count cartoon characters in order to stimulate neural activity associated with attention processing. The character count ranged randomly from 5 to 16, and the images were sufficiently large for simple viewing and counting. Each image was presented immediately after the child’s response to maintain ongoing stimulation without interruption during the recording [47]. This dataset has the potential to enhance our comprehension of the neurophysiological basis of ADHD and the creation of EEG-based tools for diagnosis or treatment.

### 3.3. Proposed ADHD-AID Tool

**Figure 3:**
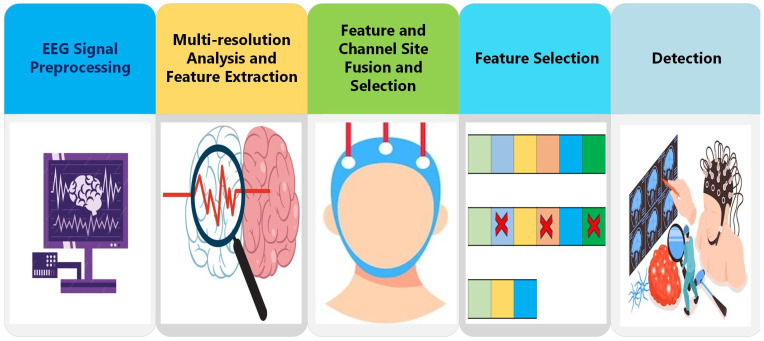
The steps of the proposed automated tool ADHD-AID.

The proposed automated detection tool named ADHD-AID has five steps, which are EEG signal pre-processing, multi-resolution analysis and feature extraction, feature and channel site fusion and selection, FS, and detection. Initially, the EEG signals are filtered and segmented. Next, several multi-resolution analyses are used to decompose the segments of the EEG signals. Furthermore, numerous feature extraction approaches are utilized to extract features from time and time–frequency domains from each EEG electrode. After that, features from the time and time–frequency domains are fused and different placement sites are investigated using a sequential forward search. Afterward, various FS approaches are applied to select a reduced set of features. Finally, five machine learning classifiers are constructed and trained to detect ADHD. Figure 3 summarizes the steps of the proposed automated tool ADHD-AID.

#### 3.3.1. EEG Signal Pre-Processing

The EEG data acquired from all electrodes are initially filtered by a band-pass IIR Butterworth filter of order 6 with a frequency range from 0.5 Hz to 60 Hz during the pre-processing phase. This frequency range is selected because it includes all of the important EEG bands (delta, theta, alpha, beta, and gamma). Afterward, a 50 Hz IIR notch filter of order 2 is applied to reduce the interference caused by the power lines. Each EEG signal is segmented into four-second intervals. The aforementioned segmented intervals have labels that are exactly the same as the EEG signals they originate from (control or ADHD). The segmentation-based approach adopted in this study is consistent with the study approach of multiple researchers who used a segmentation window size of 2–10 s in the research they performed [32,33,34].

#### 3.3.2. Multi-Resolution Analysis and Feature Extraction

In this step, three multi-resolution analysis (MRA) approaches are applied to the EEG signals including VMD, DWT, and EWT. The EEG signal is decomposed into various frequency bands using wavelet functions in DWT. Researchers can use wavelet coefficients to detect and eliminate noisy elements while maintaining brain-related activity within specific frequency bands. The author has decomposed EEG signals using one decomposition level of DWT which results in approximation and detailed coefficients. Then, the approximation coefficients which resemble the denoised EEG version are used to extract features. VMD breaks down the EEG signal into a sequence of intrinsic mode functions (IMFs) with different frequency and amplitude features, along with residuals. VMD, unlike DWT, dynamically identifies these components, possibly providing a more data-centric method for eliminating noise. Researchers can achieve a cleaner EEG signal by carefully excluding IMFs that are likely to be noise. In this study, the authors included only nine IMFs and excluded residuals to reduce noise. EWT combines the advantages of DWT and VMD. DWT breaks down the signal into wavelet packets of distinct frequencies, enabling precise noise reduction in pre-determined frequency ranges. Pre-determined wavelets may not fully capture the complexities of EEG noise. VMD accomplishes this by dynamically breaking down the signal into IMFs according to their inherent characteristics. EWT bridges the gap by employing wavelets for decomposition while integrating an adaptive sifting process comparable to VMD. This iterative process identifies and separates noise components in the IMFs, allowing for their elimination while maintaining the original brain activity in the EEG signal. EWT provides a potentially better approach for cleaning EEG data by merging the structured breakdown of DWT with the data-driven adjustment of VMD. The present study employs a maximum of nine peaks to ascertain the passbands of the EWT filter. If EWT detects fewer peaks than the given number, it will utilize the maximum number of peaks that are present. If no peaks are detected, EWT employs a level-one DWT filter bank.

**Figure 4:**
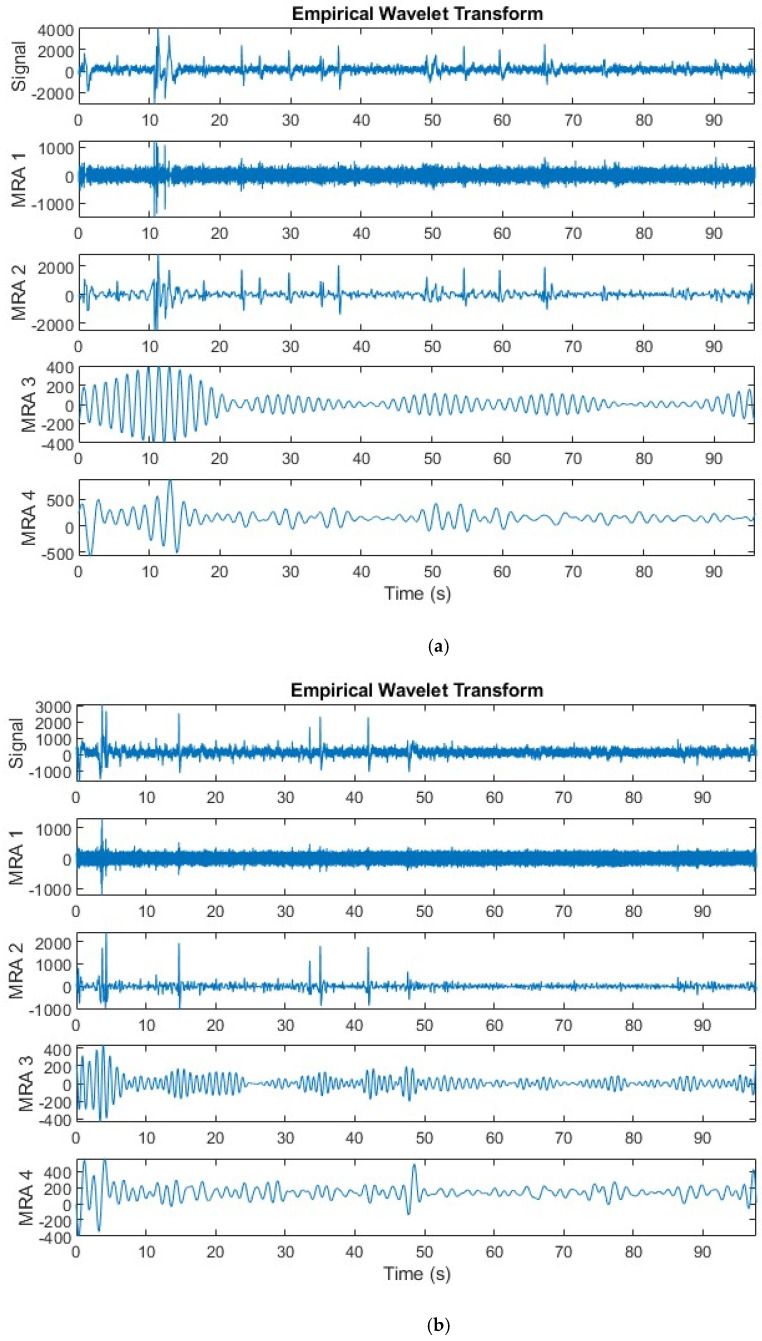

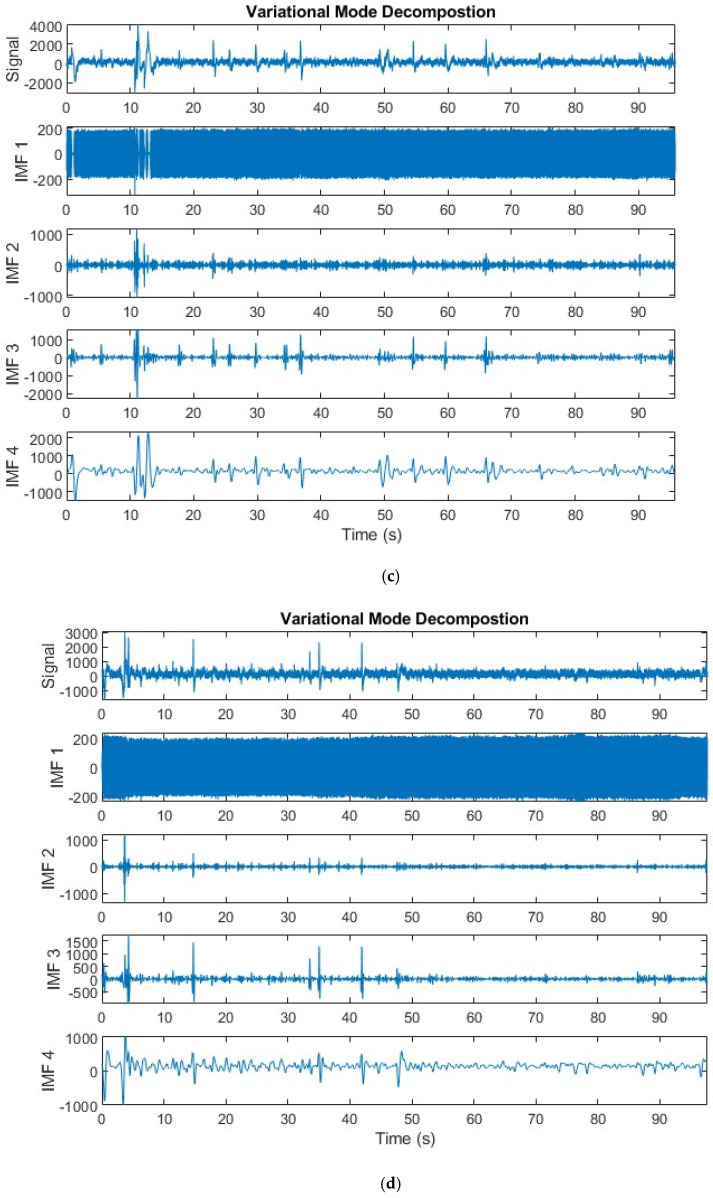
Samples of the segmented EEG signal after VMD and EWT multi-resolution analysis (MRA); (**a**) EEG signals for an ADHD patient corresponding to the first five decomposed MRA components of EWT; (**b**) EEG signals for a healthy patient corresponding to the first five decomposed MRA components of EWT; (**c**) EEG signals for an ADHD patient corresponding to the first five decomposed intrinsic mode functions (IMFs) of VMD; (**d**) EEG signals for a healthy patient corresponding to the first five decomposed IMFs of VMD.

After MRA, various complex attributes are extracted from segmented and denoised EEG signals in the time domain and their time–frequency representations using VMD, DWT, and EWT methods. To achieve this, some of the most significant prior works based on EEG signal processing were carefully examined and I subsequently determined a set of essential features. Based on the systematic review article [49] that examined and analyzed the utilization of EEG indices to measure how well individuals perform in various cognitive tasks, the common EEG indices used in the literature were selected. In addition, according to the research article [50] that assessed and contrasted different types of EEG indices for the identification of ADHD, those EEG indices that were capable of diagnosing ADHD were employed in this study. An attempt to cover all significant feature types during that process was conducted. These features include nonlinear features, band-power features, entropy-based features, and statistical features. These feature extraction approaches are summarized in Table 2. Samples of the segmented EEG signal after VMD and EWT MRA are displayed in Figure 4. Note that the frequency boundaries are delta (1–4 Hz), theta (4–8 Hz), alpha (8–12 Hz), beta (12–30 Hz), and gamma (30–60 Hz) [4].

#### 3.3.3. Feature and Channel Site Fusion and Selection

In this step, features extracted from EEG signals in time and time–frequency domains using VMD, DWT, and EWT methods are concatenated. Next, different electrode sites are examined in a sequential forward search strategy. First, each electrode placement site is ranked according to the detection accuracy achieved in the detection step. After that, the site with the highest accuracy is set as the initial site set. Afterward, subsequent electrode placement sites are added iteratively according to their rank. The search terminates when all electrode placement sites are processed.

#### 3.3.4. Feature Selection

In this step, the attributes of the chosen site set are subjected to an FS procedure in order to determine a smaller set of attributes that influence detection efficiency. FS refers to a collection of computational techniques that aim to identify the most appropriate features out of the original set of features. FS is an effective method for dealing with extremely multi-dimensional information since it can reduce feature dimensions and redundancy, which can help with issues such as overfitting models. FS can also shorten the period of the learning/detection time and simplify classifiers’ complexities. In FS, only those attributes that satisfy pre-defined criteria or optimize specific computation methods are determined and selected by FS algorithms. In this study, three FS approaches are employed to choose a reduced number of features. These FS approaches include analysis of variance (ANOVA), Chi-squared (Chi^2^), and Kruskal–Wallis (KW).

**ANOVA** [59] is a method used to analyze empirical information where a number of attributes are determined according to different conditions that are recognized by a single attribute or further classification of attributes. The one-way ANOVA technique can be utilized for evaluating null hypotheses based on similar averages across various populations. ANOVA can provide information regarding whether all averages have equal significance or if there appears to be a disparity between the averages of various groups.

**Chi^2^** is a popular FS approach that determines the deviation from the anticipated distribution if an attribute’s occurrence is independent of its classification label [60].

**Kruskal–Wallis (KW)** is a method that calculates the score *S* by determining whether the median of more than one class label is equal. Chosen features have discriminatory ability. The attribute can only be chosen if it includes discriminatory information, which is indicated by a score *S* close to “0” [61].

#### 3.3.5. Detection

The final step of the ADHD-AID automated tool is detection. In this step, five machine learning classifiers are employed including cubic-SVM (C-SVM), quadratic SVM (Q-SVM), medium Gaussian SVM (M-SVM), k-nearest neighbor (kNN), and ANN. The effectiveness of the classification algorithms is evaluated using k-fold validation (*k* = 10), in which the data are divided at random into *k* portions for both training and testing. The classification algorithm is created using *k*-1 portions during the training process, and the remaining *k* is utilized for testing where an estimation of the model’s accuracy is achieved. This procedure is repeated, where all time distant *k*-1 folds are utilized for testing, and the remaining *k* for testing.

## 4. Parameter Setting

For VMD and EWT, the number of intrinsic mode functions are nine. The Symlets-5 mother wavelet is employed for DWT, where the number of decomposition levels are one and other parameters are kept in their default values in MATLAB 2022a. Note that *k* = 3 and the Euclidean distance metrics are used for the k-NN classifier, whereas for the ANN, two hidden layers are employed. Each hidden layer has 10 neurons with a rectified linear unit activation function. For the SVM classifier, cubic, quadratic, and medium Gaussian kernels are used. The box constraint level is one.

## 5. Detection Results

This section initially introduces the results of the five classification models fed with features obtained from the segmented EEG signals in time and time–frequency domains extracted by VMD, DWT, and EWT methods for all of the channels. Next, the results achieved using the concatenated features from the multiple domains are presented in parallel with those attained using different electrode location sites. After that, the detection results of the examination of several channel site fusions utilizing a sequential search strategy are demonstrated. Finally, the performance metrics calculated after the FS approaches are illustrated.

### 5.1. Multi-Domain Feature Extraction Results

This section compares the results of the five classifiers achieved using features extracted from segmented multi-dimensional EEG signals in the time domain and the time–frequency domain. The time–frequency representations are obtained by using VMD, DWT, and EWT multi-resolution analysis methods. The comparison of results is displayed in Table 3. As can be seen in Table 3, the time domain features achieved peak accuracy of 97.9%, 98.5%, 96.3%, 97.7%, and 96.4% for the Q-SVM, C-SVM, M-SVM, k-NN, and ANN classifiers. Whereas for DWT-based features, the accuracy is slightly lower than that of the time domain features and equal to 97.7%, 98.2%, 96.2%, 97.9%, and 95.5% for the Q-SVM, C-SVM, M-SVM, k-NN, and ANN classifiers. In addition, the accuracy attained using EMT-based features is 96.8%, 97.9%, 95.6%, 98.1%, and 94.9% for the Q-SVM, C-SVM, M-SVM, k-NN, and ANN classifiers, and for the VMD-based features, the accuracy is 96.3%, 97.3%, 94.5%, 97.1%, and 94.1% for the same classifiers. These accuracies indicate that the time domain and DWT-based features have comparable performance and are higher than those obtained by EWT and VMD.

### 5.2. Electrode Placement Site Results

The time and time–frequency domain features (VMD, DWT, and EMT) are merged and then different electrode locations are investigated. The results of the classification models fed with fused time and time–frequency domain features for each electrode placement are presented in Table 4. The results in the table indicate that the combined features of frontal location achieved the highest accuracy, 91.2%, 93.5%, 90.1%, 95.1%, and 88.8% for the Q-SVM, C-SVM, M-SVM, k-NN, and ANN classifiers. Afterward, those features obtained by the temporal site reached an accuracy of 89.5%, 91.6%, 87.5%, 92.4%, and 86.9% for the Q-SVM, C-SVM, M-SVM, k-NN, and ANN classifiers. Subsequently, parietal position features accomplished an accuracy of 88.1%, 90.6%, 87.3%, 92.7%, and 85.9% for the Q-SVM, C-SVM, M-SVM, k-NN, and ANN classifiers. Following that, the accuracy attained by features of central electrode position was 84.3%, 87.4%, 83.6%, 88.3%, and 82.0% for the Q-SVM, C-SVM, M-SVM, k-NN, and ANN classifiers. Afterward, the occipital electrode site’s features reached an accuracy of 83.5%, 84.2%, 82.3%, 86.1%, and 80.6% for the Q-SVM, C-SVM, M-SVM, k-NN, and ANN classifiers. According to the detection accuracies achieved by each electrode position, these locations have been ranked in descending order and then investigated using a sequential forward strategy to search for the best combination of sites that influence the performance of ADHD-AID.

### 5.3. Electrode Site Selection Results

The results of investigating fusing electrode positions using a sequential forward strategy are demonstrated in this section. Table 5 shows the detection accuracy for the combination of electrode sites. It can be observed from Table 5 that the detection accuracy starts to increase by adding more electrode positions. This is because initially, with the combined features of frontal electrodes alone, the accuracy is 91.2%, 93.5%, 90.1%, 95.1%, and 88.8% for the Q-SVM, C-SVM, M-SVM, k-NN, and ANN classifiers. This accuracy keeps on increasing by considering more electrode locations until it reaches 98.2%, 98.8%, 97.1%, 98.5%, and 96.6% using the same classifiers fed with the fused features of the temporal plus frontal plus parietal plus central plus occipital plus pre-frontal electrode sites. The results indicate that employing more electrode sites has a positive impact on the detection accuracy at the cost of an increase in the feature space dimensionality.

### 5.4. Feature Selection Results

As mentioned earlier, the addition of more electrode locations improves the performance; however, it also makes the dimension of the feature space huge, which increases the complexity and training duration of the classification models. Thus, in this study, three FS approaches are employed to reduce the dimensionality of feature space. The results of the three FS methods are compared and displayed in Table 6. For the Chi^2^ FS approach, the highest accuracies (98.9% and 98.8%) are achieved using C-SVM and k-NN classifiers with 1500 features, followed by the Q-SVM (98.4%) with 2000 features. Subsequently, the M-SVM and ANN achieve an accuracy of 97.3% and 96.8% utilizing 2000 and 1500 features, respectively, whereas for the ANOVA FS approach, the peak accuracies (99.1% and 98.6%) are attained by the C-SVM and k-NN with 1000 features. Following that, the Q-SVM, M-SVM, and ANN classifiers reach an accuracy of 98.4%, 97.3%, and 96.9% using 2000, 2000, and 1000 features, respectively. Meanwhile for the KW FS approach, the C-SVM and k-NN accomplish the maximum accuracy of 99% and 98.7% using 1000 features. Next, the Q-SVM, M-SVM, and ANN classifiers attain an accuracy of 98.4%, 97.5%, and 97.1% using 1500 features. Note that the accuracies accomplished with the three FS approaches are greater than those displayed in Table 5 but with a lower number of features obtained from all features of the 19 electrodes (2508 features).

**Figure 5:**
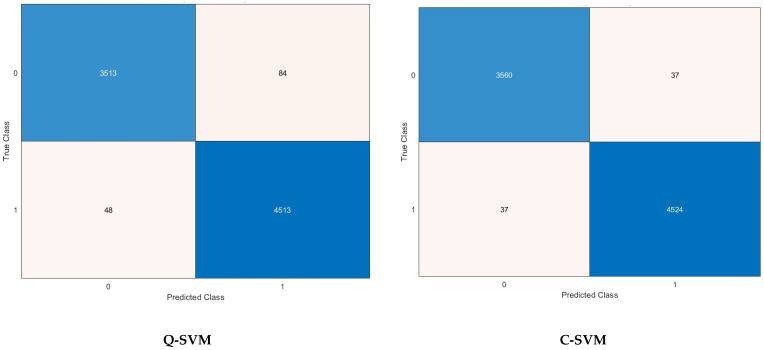

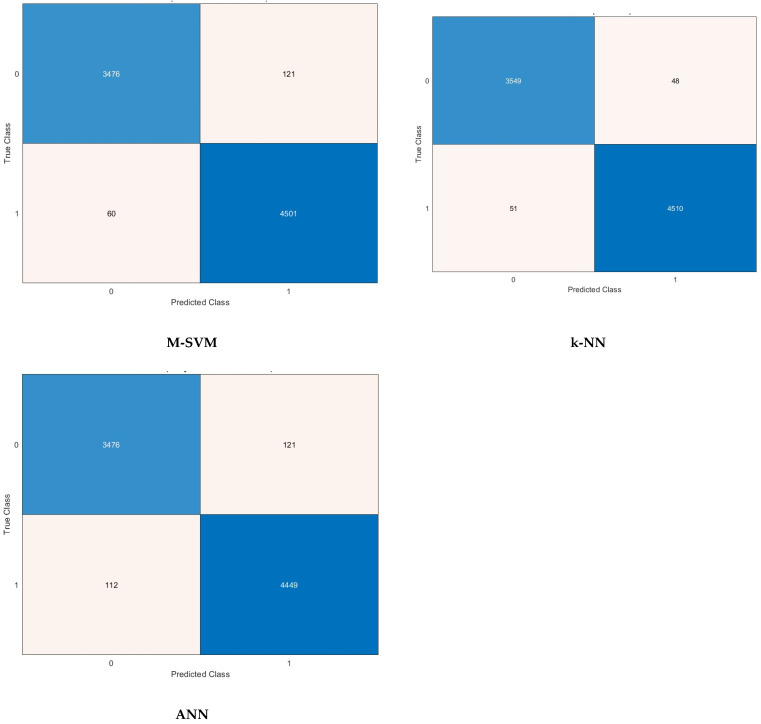
Confusion matrices after the FS step for the best scenario for each classifier.

Other performance metrics including sensitivity, precision, specificity, MCC, and F1-score are further calculated for the highest scenario achieved for each classification model in Table 6. It can be noticed that the sensitivity, specificity, precision, F1-score, and MCC for the C-SVM and M-SVM are (98.9% and 96.6%), (99.2% and 98.7%), (98.9% and 98.3%), (98.9% and 97.5%), and (98.2% and 95.5%) using the 1000 features selected by the ANOVA FS approach. Meanwhile, for Q-SVM and ANN, the 1500 features chosen using the KW FS method lead to a sensitivity, specificity, precision, F1-score, and MCC of (97.7% and 96.6%), (98.9% and 97.5%), (98.7% and 96.9%), (98.2% and 96.8%), and (96.7% and 94.2%), respectively. However, for k-NN, the sensitivity, specificity, precision, F1-score, and MCC achieved utilizing the 1500 picked by the Chi^2^ FS approach are 98.7%, 98.9%, 98.6%, 98.6%, and 97.5%, respectively. The confusion matrices employed to calculate these performance metrics achieved after the FS step for the best scenario for each classification model mentioned before are shown in Figure 5.

It can be noted from Table 7 that Q-SVM outperforms all other FS methods and feature counts in terms of achieving the highest F1-score, with scores ranging from 97.5% to 98.2%. C-SVM excels when combined with ANOVA FS, yielding F1-scores ranging from 98.5% to 98.9%. Its performance diminishes marginally when compared to other feature selection methods. The M-SVM classifier has the lowest F1-score compared to the other two classifiers, with F1-scores ranging from 97.1% to 97.5%. k-NN consistently performs well, with F1-scores of approximately 98.6% across various FS methods and feature counts. ANN exhibits the worst performance among all classifiers, with F1-scores varying from 96.3% to 96.8%.

Table 7 shows that all classifiers demonstrated high precision ranging from 96.2% to 98.9% and specificity ranging from 98.2% to 99.2%, suggesting a strong capability to accurately distinguish between positive and negative cases.

Sensitivity is high for all classifiers, ranging from 96.5% to 99.2%, except for M-SVM, which has a slightly lower sensitivity ranging from 96.5% to 97.0%. Some positive cases may go undetected by M-SVM. It can be concluded from Table 7 that Q-SVM appears to be the most resilient classifier when tested with various FS methods and feature quantities. Utilizing ANOVA FS with C-SVM could enhance accuracy, although other classifiers demonstrate similar performance even without FS.

**Figure 6:**
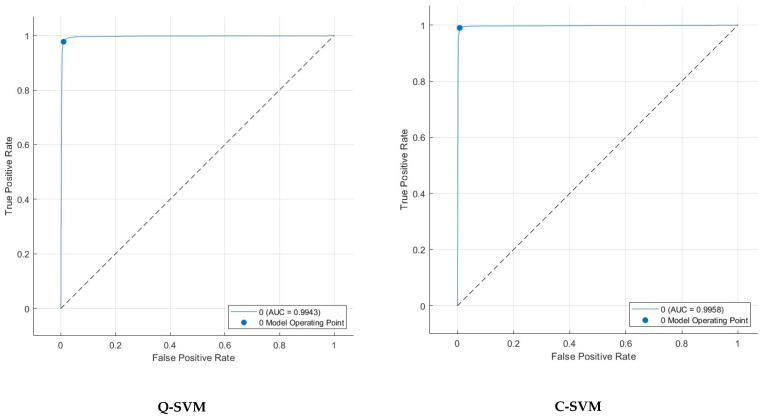

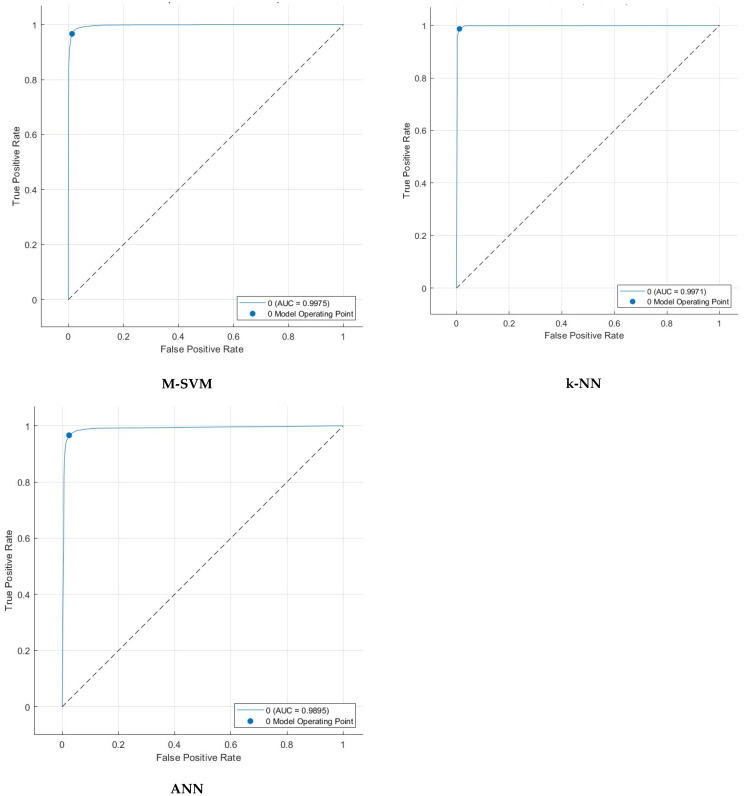
ROC curves and associated AUC after FS step for the best scenario for each classifier.

A confusion matrix is a key tool for assessing the effectiveness of classification models in machine learning. The tabulated structure displays a comparison between the model’s predicted classes and the true (actual) classes. The matrix comprises essential components which are TP, TN, FP, and FN. Comprehending the outcomes of a confusion matrix is essential for assessing the capabilities and limitations of a classification model while making well-informed decisions for enhancement. For Q-SVM, the TN and TP are 3513 and 4513, while FN and FP are 48 and 84. For C-SVM, the TN and TP are 3560 and 4524, while FN and FP are 37 and 37. In the case of the M-SVM classifier, the TN and TP are 3476 and 4501, while FN and FP are 60 and 121. Whereas in the case of k-NN, the TN and TP are 3549 and 4510, while FN and FP are 51 and 48. For ANN, the TN and TP are 3476 and 4449, while FN and FP are 112 and 121. The receiving operating characteristic (ROC) curves for the best scenario for each classifier are plotted and displayed in Figure 6. Furthermore, the area under the ROC curves (AUC) is determined and displayed in Figure 6. The ROC curve illustrates the balance between a classifier’s true positive rate and false positive rate at different classification thresholds. The AUC measures the model’s capacity to differentiate between classes. An AUC of 1.0 signifies an ideal classifier that can perfectly distinguish between positive and negative instances, whereas an AUC of 0.5 suggests a model that performs no better than random chance. The ROC results show outstanding discriminating capabilities for all classifiers, with AUC values very near to 1.0. The M-SVM classifier obtained an AUC of 0.9975, demonstrating superior capability in distinguishing between positive and negative instances at different classification thresholds. Closely behind are C-SVM with an AUC of 0.9958 and k-NN with an AUC of 0.9971, both showing exceptional classification performance. Q-SVM and ANN demonstrate strong performance, with AUC values of 0.9943 and 0.9895, respectively, slightly below the other three classifiers. The results indicate that all models could be useful in a diagnostic or classification scenario where accurate discrimination is crucial.

## 6. Discussion

This study introduces an ML-based tool for automatically detecting ADHD. The present study employs various MRA techniques to analyze EEG waves and eliminate background noise in order to address the limitations identified in the literature. In the research procedure, thirty features are retrieved from the time and time–frequency domains to detect ADHD, comprising nonlinear features, band-power features, entropy-based features, and statistical features. This study also examines the optimal EEG location of electrodes to identify ADHD, identifying the location configurations that have the most significant effect on detection accuracy. Various feature selection strategies are utilized to determine which features are most likely to impact the diagnosis of ADHD, thereby decreasing the complexity and training duration of the classification process.

The MRA results indicated that features obtained through DWT are more accurate compared to those acquired through empirical wavelet transform (EWT) and variational mode decomposition (VMT). The time domain features achieved similar accuracy to the DWT features. By integrating time domain features with three multi-resolution analysis methods (DWT, EWT, and VMD) and by examining various electrode positions, it was observed that the frontal position significantly influences performance more than other electrode locations. When seeking the optimal electrode location, combining all electrode sites was found to have the greatest impact on performance, despite the drawback of increasing feature space dimensions and training complexity. The results showed that using various feature selection approaches led to a reduction in feature dimensionality, resulting in improved detection accuracy.

**Figure 7:**
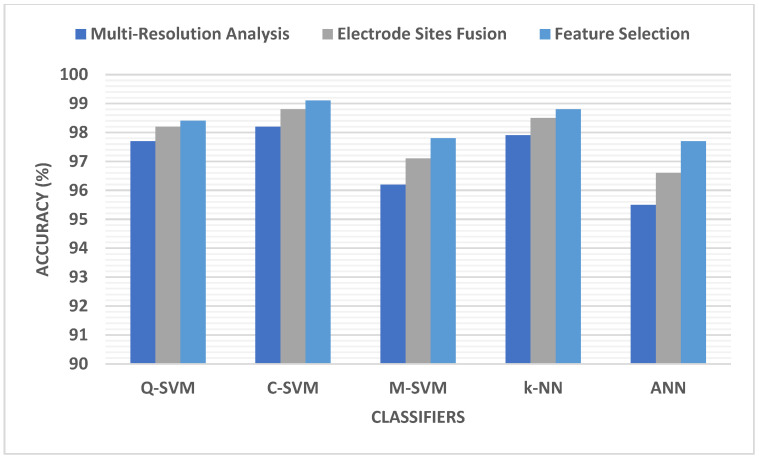
Detection accuracy comparison between the highest performance achieved during multi-resolution analysis of the electrode site fusion.

Figure 7 shows a comparison between the peak performance reached in MRA and the resulting fusion of electrode sites. The data confirm that accuracy improves after combining electrode sites and merging features from multiple multi-resolution analyses. The accuracy was enhanced through multiple feature selection approaches, resulting in a reduced number of features. The final accuracies obtained after the FS step are 98.4% for Q-SVM, 99.1% for C-SVM, 97.8% for M-SVM, 98.8% for k-NN, and 97% for ANN classifiers. These accuracies were obtained by utilizing 1500 features for Q-SVM, k-NN, and ANN, using the KW FS technique for Q-SVM and ANN, and the Chi2 FS method for k-NN. On the other hand, the accuracy values for C-SVM and M-SVM were acquired using 1000 features with the ANOVA FS method.

### 6.1. Comparative Analysis

In order to confirm the competitive capabilities of the proposed ADHD-AID automated tool, the performance measurements achieved by ADHD-AID are compared with recent related studies based on the same ADHD IEEE Dataport dataset. The comparison is illustrated in Table 8. It can be concluded from Table 8 that the proposed ADHD-AID tool has a significant competing ability. This is obvious as ADHD-AID has an outstanding performance compared to existing studies for ADHD detection. The accuracy, sensitivity, specificity, F1-score, and MCC achieved by ADHD-AID are 0.991, 0.989, 0.992, 0.989, and 0.982, which are greater than those obtained by previous studies. The reason for this is that in contrast to other studies based on a larger number of features [21,24], ADHD-AID extracts thirty features including nonlinear features, band-power features, entropy-based features, and statistical features. Furthermore, ADHD-AID acquires these features from time and time–frequency domains of several multi-resolution analysis approaches such as VMD, DWT, and EWT, which is not the case in previous studies [21,23,24,26,36]. In addition, it employs an FS approach to select the influential features that impact performance, in contrast to other studies [23,31,36,62].

### 6.2. Limitations and Upcoming Works

This study has a number of constraints. The main restriction is the small number of collected samples in the dataset employed. Because of the restrictions on the clinical gathering of data, there were limits on the dataset’s size and the number of subjects, which make the findings less convincing. Another limitation is due to the unavailability of public datasets for ADHD detection among children. This study only utilized one public dataset. A further drawback is that considering the diversity of ADHD individuals [63], the subgroup of children with ADHD had not been considered. By employing a sizable and evenly matched sample of adolescents, more thorough analyses will be carried out to validate this tool. The present study did not employ deep learning, explainable AI, and uncertainty quantification approaches. Future work will take into account deep learning techniques [64] including LSTM and CNN [65], explainable AI approaches [66] such as Gradient-weighted Class Activation Mapping (Grad-CAM) [67], as well as uncertainty quantification approaches like the Bayesian method. This study also did not use ensemble techniques [68], so further work will include ensemble techniques instead of using individual models. In the upcoming work, the proposed model can be applied to detect other diseases and can be involved in more EEG applications such as emotion recognition and personal identification.

EEG features are currently improbable as an independent diagnostic method for ADHD. EEG shows potential for studying the neurophysiological mechanisms of ADHD, similar to how structural or functional brain imaging is used, without replacing a thorough clinical evaluation. The author firmly believes that a comprehensive clinical assessment following DSM criteria, which includes input from parents, teachers, and possibly other experts, is essential for diagnosing ADHD. However, the EEG criteria-based ML tool was developed as an adjunct, not as the sole determinant in diagnosing ADHD. The proposed tool is an aiding tool that aids physicians by combining their clinical expertise with EEG to accomplish accurate detection.

It is worth mentioning that the EEG is not the best approach for detecting function localization. Future work will consider using magnetic resonance imaging (MRI), functional MRI (fMRI), diffusion tensor imaging, or positron emission tomography (PET) [69,70]. Also, this study employed only 19 EEG electrodes. Future work will consider using more electrodes for a comprehensive independent analysis of the components and to exclude the biological artifacts. Furthermore, EMG, oculography, and ECG will be performed. Since the EMG of the frontal muscles of the scalp tone is increased in patients with ADHD, it is very important to consider EMG in upcoming investigations.

Additionally, not providing the frequency analysis of individual alpha peaks is one of the limitations of this study since the individual frequency of the alpha peak is the key to determining the boundaries of other frequency ranges. Moreover, it is known that the individual alpha peak frequency in an eyes-closed condition is decreased in patients with ADHD [71,72]. Upcoming work will consider performing frequency analysis of individual alpha peaks and studying the individual alpha peak frequency in eyes-closed cases for patients with ADHD. Also, the frequency of the alpha peak, the level of neuronal activation, and the EMG tone of the frontal muscles depend on the hormonal state of women; for example, the frequency is higher in the luteal phase (progesterone phase) and lower in the menstrual, follicular, and pre-menstrual phases. Accordingly, it is necessary to take this fact into account when assessing EEG activity and when conducting machine learning. Unfortunately, the present study did not investigate the gender factor when evaluating EEG activity and when operating machine learning, which will be taken into consideration in future work. Furthermore, the potential future extensions of this work include the incorporation of EEG channel selection methods and the detection of children’s neurodevelopmental conditions aside from ADHD. Upcoming work will also conduct experiments employing the leave-one-subject-out cross-validation procedure to look for subject-wise accuracy levels, taking into account long EEG signals.

More EEG data will be analyzed, focusing on features associated with ADHD pathophysiology. This will enable us to recognize patterns in the cleaned signals that could be more pertinent to comprehending the fundamental mechanisms of ADHD. Furthermore, a more thorough analysis will be conducted to compare the ADHD group with the control group. This will entail statistically comparing the identified features to determine if the denoising process uncovered any notable differences in brain activity patterns associated with ADHD. In addition, upcoming research will concentrate on interpreting the results in relation to the current understanding of ADHD and EEG. This will connect technical analysis with its potential implications for real-world clinical practice.

## 7. Conclusions

This study presented an aiding tool called ADHD-AID that assists physicians by integrating their clinical experience with EEG to achieve accurate detection of ADHD. Contrary to previous research that obtained few feature extraction approaches to extract features from a single domain, this study employed a large variety of features. ADHD-AID obtained these features from time domains as well as various multi-resolution analysis methods including VMD, DWT, and EWT. Furthermore, ADHD-AID explored the impact of different electrode locations on ADHD detection accuracy. It also investigated the influence of fusing features from multiple domains and different sites on the detection performance. The raw EEG signals do not offer a lot of useful discriminating information. Consequently, thirty attributes are retrieved in this study including nonlinear features, band-power features, entropy-based features, and statistical features. The outcome of this research demonstrated that the frontal area is essential for identifying ADHD in children. In addition, the results proved that merging features from the time domain and several time–frequency domain approaches can boost performance. The findings also showed that combining more electrode sites can enhance performance at the cost of an increase in feature space dimension. Thus, when employing multiple FS approaches including Chi^2^, ANOVA, and KW to reduce the huge dimensionality, the results indicated that the three FS approaches were capable of improving performance while lowering the number of features employed to train the classification model, which consequently reduced training complexity and duration. The results proved that the suggested ADHD-AID tool has a remarkable capacity for learning to detect the fundamental variation in ADHD-affected kids, and this finding offers a chance for comprehending the possible mechanism of ADHD and for developing an exceptionally reliable secondary detection system.

## Figures and Tables

**Table 1:** Summary of the literature on ADHD recognition along with the limitations of each study.

Article	Dataset	Feature Extraction	Feature Selection	Models	Accuracy	Limitations
[21]	IEEE Dataport [38] 60 Healthy 61 ADHD	A total of 15 features including power, energy, entropy, and statistical-based features obtained from EMD	GA	ANN	96.16%	Extracted features from a single domain. Employed individual classification models. Not very high accuracy. Did not search for the best electrode placement on the skull.
[22]	IEEE Dataport [38] 60 Healthy 61 ADHD	A total of 41 statistical features from the fifth mode of VMD-HT	N/A	EBM	99.81%	Extracted features from a single domain. Obtained only nonlinear and statistical features. Did not employ FS. High complexity of the models due to large feature space.
[25]	Private Dataset 15 Healthy 18 ADHD	A total of 15 connectivity-based features from different modes of ITD	N/A	Bagging Trees	99.46%	Extracted features from a single domain. Did not employ FS. Depended on a private dataset. Did not search for the best electrode placement on the skull.
[26]	IEEE Dataport [38] 60 Healthy 61 ADHD	ECMs using dPTE	GA	ANN	89.1%	Extracted features from a single domain. Employed one method for feature extraction. Did not search for the best electrode placement on the skull. Employed individual classification models. Low accuracy.
[23]	IEEE Dataport [38] 60 Healthy 61 ADHD	Employed EEG signals directly to training models	N/A	Adaboost	84%	Did not employ feature extraction. Did not perform FS. Low accuracy.
[6]	Private Dataset 50 ADHD 58 Healthy	PSD + entropy features + bi-spectral features	mRMR	SVM	84.59%	It depended on a private dataset. Did not search for the best electrode placement on the skull. Employed individual classification models. Low accuracy.
[24]	IEEE Dataport [38] 60 Healthy 61 ADHD	A total of 10 statistical, power spectral density, and entropy-based features from the time domain	PCA	SVM	94.2%	Features extracted from the time domain only. Few features were extracted. Employed individual classification models. Did not search for the best electrode placement on the skull. Low accuracy.
[39]	Private Dataset 12 ADHD 12 Healthy	Linear univariate and multi-variate features + nonlinear univariate and multi-variate features	N/A	SVM	99.58%	Extracted frequency from a single domain. Did not employ FS. Employed individual classification models. High feature dimension. Did not search for the best electrode placement on the skull.
[31]	IEEE Dataport [38] 60 Healthy 61 ADHD	N/A	N/A	LSTM+GRU	95.33%	Studied features from the temporal domain only. High complexity of the classification models. Low accuracy. Did not search for the best electrode placement on the skull. Depended on a private dataset relying on a low number of subjects.
[32]	Private Dataset [40] 15 Healthy 15 ADHD	N/A	N/A	CNN	97.47%	Studied features from spatial domain only. High complexity of the classification models. Not very high accuracy. Did not search for the best electrode placement on the skull. Employed individual classification models. Employed a private dataset relying on a low number of subjects.
[33]	Private Dataset 51 Healthy 50 ADHD	A total of 13 connectivity-based features from the brain network	N/A	CNN	94.67%	Obtained features from a single domain. Used one feature extraction approach. Huge feature dimension. The complexity of the classification model was high. Did not employ FS. Employed individual classification models. Used a private dataset. Did not search for the best electrode placement on the skull.
[34]	IEEE Dataport [38] 46 ADHD 45 Healthy	Dynamic connectivity tensor	N/A	Conv-LSTM	99.34%	Used one feature extraction approach. The complexity of the classification model was high. Did not employ feature selection. Did not search for the best electrode placement on the skull.
[35]	Private Dataset 44 Healthy 100 ADHD	N/A	N/A	EEGNet	83%	Low accuracy. Employed a single classification model. The complexity of the classification model was high. Did not employ feature selection. Did not search for the best electrode placement on the skull. Dataset was private. Employed individual classification models. Unbalanced dataset.
[36]	IEEE Dataport [38] 60 Healthy 61 ADHD	VMD+RLMD	N/A	CNN	95.24%	Obtained features from a single domain. Did not perform feature selection. Did not search for the best electrode placement on the skull. High complexity of the classification model.
[37]	IEEE Dataport [38] 30 Healthy 31 ADHD	PSD frequency features using FT	N/A	CNN	94.52%	Obtained features from a single domain. Did not perform feature selection. Did not search for the best electrode placement on the skull. High complexity of the classification model.

N/A: Not applicable.

**Table 2:** A list of the feature extraction approaches.

Hjorth Activity [51]	Renyi Entropy [52]	Skewness [53]
Hjorth Mobility [51]	Shanon Entropy [52]	Kurtosis [53]
Hjorth Complexity [51]	Log Energy Entropy [54]	Auto Regressive Model [55]
Log Root Sum of Sequential Variation [51]	Tsallis Entropy [52]	Band-Power Alpha
Mean Curve Length [56]	First Difference [57]	Band-Power Beta
Mean Energy [56]	Second Difference [57]	Band-Power Theta
Mean Teager Energy [56]	Normalized First Difference [57]	Band-Power Gamma
Median [56]	Normalized Second Difference [57]	Band-Power Delta
Minimum [56]	Variance [58]	Ratio Band-Power Alpha Beta
Maximum [56]	Standard Deviation [58]	Arithmetic Mean [56]

**Table 3:** Detection accuracy (%) for time and time–frequency domain features (VMD, DWT, and EMT).

Method	Q-SVM	C-SVM	M-SVM	k-NN	ANN
Time	97.9	98.5	96.3	97.7	96.4
DWT	97.7	98.2	96.2	97.9	95.5
EWT	96.8	97.9	95.6	98.1	94.9
VMD	96.3	97.3	94.5	97.1	94.1

**Table 4:** Detection accuracy (%) for combining time and time–frequency domain features at different electrode locations.

Electrode Locations	Q-SVM	C-SVM	M-SVM	k-NN	ANN
Pre-Frontal	82.9	86.1	81.6	86.9	81.0
Frontal	91.2	93.5	90.1	95.1	88.8
Central	84.3	87.4	83.6	88.3	82.0
Parietal	88.1	90.6	87.3	92.7	85.9
Temporal	89.5	91.6	87.5	92.4	86.9
Occipital	83.5	84.2	82.3	86.1	80.6

**Table 5:** Detection accuracy (%) for investigating fusing electrode positions using a sequential forward strategy.

Electrode Locations	Q-SVM	C-SVM	M-SVM	k-NN	ANN
Frontal	93.5	90.1	95.1	88.8	91.2
Temporal + Frontal	95.7	97.0	94.4	97.2	93.7
Temporal + Frontal + Parietal	97.5	98.1	95.7	97.9	95.4
Temporal + Frontal + Parietal + Central	97.4	98.4	96.5	98.1	95.9
Temporal + Frontal + Parietal + Central + Occipital	97.8	98.6	96.9	98.2	96.0
Temporal + Frontal + Parietal + Central + Occipital + Pre-Frontal	98.2	98.8	97.1	98.5	96.6

**Table 6:** Detection accuracy (%) after the FS step using the three FS methods.

Features	Q-SVM	C-SVM	M-SVM	k-NN	ANN
Chi^2^					
1000	98.0	98.7	96.6	98.7	96.6
1500	98.3	98.9	96.9	98.8	96.8
2000	98.4	98.9	97.3	98.7	96.8
ANOVA					
1000	98.3	99.1	97.8	98.6	96.9
1500	98.3	98.9	97.8	98.5	96.7
2000	98.4	98.8	97.3	98.6	96.4
Kruskal–Wallis (KW)					
1000	98.1	99.0	97.0	98.7	96.7
1500	98.4	99.0	97.5	98.7	97.1
2000	98.4	98.9	97.3	98.6	96.4

**Table 7:** Performance metrics (%) after the FS step for the best scenario for each classifier.

	FS Method	Features Number	Sensitivity	Specificity	Precision	F1-Score	MCC
Q-SVM	KW	1000	97.2	98.8	98.5	97.8	96.2
Q-SVM	KW	1500	97.7	98.9	98.7	98.2	96.7
Q-SVM	KW	2000	97.5	98.8	98.5	98.0	96.4
C-SVM	ANOVA	1000	98.9	99.2	98.9	98.9	98.2
C-SVM	ANOVA	1500	98.6	99.1	98.9	98.7	97.7
C-SVM	ANOVA	2000	98.5	99.0	98.7	98.6	97.5
M-SVM	ANOVA	1000	96.6	98.7	98.3	97.5	95.5
M-SVM	ANOVA	1500	97.0	98.5	98.1	97.5	95.6
M-SVM	ANOVA	2000	96.5	98.2	97.6	97.1	94.8
k-NN	Chi^2^	1000	98.3	98.9	98.6	98.6	97.2
k-NN	Chi^2^	1500	98.7	98.9	98.6	98.6	97.5
k-NN	Chi^2^	2000	98.6	98.9	98.6	98.6	97.4
ANN	KW	1000	96.4	96.9	96.2	96.3	93.3
ANN	KW	1500	96.6	97.5	96.9	96.8	94.2
ANN	KW	2000	96.4	97.0	96.3	96.4	93.5

**Table 8:** Comparative performance analysis with recent related studies based on the same ADHD IEEE Dataport dataset.

Article	Feature Extraction	Feature Selection	Models	Accuracy	Sensitivity	Specificity	F1-Score	MCC
[21]	A total of 15 features including power, energy, entropy, and statistical-based features obtained from EMD	GA	ANN	96.16%	-	-	96.32%	92.0%
[26]	ECMs using dPTE	GA	ANN	89.1%	-	-	-	-
[23]	Employed EEG signals directly with training models	N/A	Adaboost	84%	96.0%	70.0%	-	-
[24]	A total of 10 statistical, power spectral density, and entropy-based features from time domain	PCA	SVM	94.2%	-	-	-	-
[31]	N/A	N/A	LSTM+GRU	95.33%	96.20%		95.80%	
[36]	VMD + RLMD	N/A	CNN	95.24%	-	-	-	-
[62]	Cross-recurrence plots + Welch power spectral distribution	N/A	N/A	97.24%	97.0%	94.0%	-	-
Proposed ADHD-AID	A total of 30 features (nonlinear features, band-power features, entropy-based features, and statistical features) from time and time–frequency domains of VMD, DWT, and EWT	ANOVA	C-SVM	99.10%	98.9%	99.2%	98.9%	98.2%

N/A: Not applicable.

## Data Availability

The dataset for children with ADHD can be found at the following link: https://ieee-dataport.org/open-access/eeg-data-adhd-control-children (Accessed: 1 May 2023).

## Abbreviations

**AI**	Artificial intelligence
**ADHD**	Attention deficit hyperactivity disorder
**ANN**	Artificial neural networks
**ANOVA**	Analysis of variance
**Chi2**	Chi squared
**Conv-LSTM**	Convolutional long short-term memory
**CNN**	Convolutional neural network
**C-SVM**	Cubic support vector machine
**dPTE**	Directed phase transfer entropy
**DWT**	Discrete wavelet transform
**EBM**	Explainable boosted machine
**ECM**	Effective connectivity matrices
**EEG**	Electroencephalography
**EMD**	Empirical mode decomposition
**EWD**	Empirical wavelet transform
**EVD**	Empirical variational decomposition
**FFT**	Fast Fourier transform
**fMRI**	Functional magnetic resonance imaging
**FN**	False negative
**FP**	False positive
**FS**	Feature selection
**GA**	Genetic algorithm
**Grad-CAM**	Gradient-weighted Class Activation Mapping
**GRU**	Gated recurrent network
**HT**	Hilbert transform
**ITD**	Intrinsic time-scale decomposition
**k-NN**	k-nearest neighbors
**KW**	Kruskal–Wallis
**LRM**	Layer-wise Relevance Propagation
**LSTM**	Long short-term memory
**MCC**	Mathew correlation coefficient
**ML**	Machine learning
**MRA**	Multi-resolution analysis
**MRI**	Magnetic resonance imaging
**mRMR**	Minimum redundancy and maximal relevance
**M-SVM**	Medium Gaussian support vector machine
**PCA**	Principal component analysis
**Q-SVM**	Quadratic support vector machine
**RLMD**	Robust local mode decomposition
**SVM**	Support vector machine
**TN**	True negative
**TP**	True positive
**VMD**	Variational mode decomposition
